# Obituary Notices

**Published:** 1837

**Authors:** 


### Obituary Notices.

*Death of Dr. Physick.*—We have just received intelligence of the death of this distinguished surgeon and physician, at the age of seventy years. In our next number, we shall avail ourselves of some one of the biographical memoirs, to which his native city will undoubtedly give birth, to make our readers acquainted with his life and character. At present, as our limits are full, we can only say, that we had the pleasure, 32 years ago, of attending the first course of Lectures on Surgery, which he delivered after his appointment into the University of Pennsylvania, of which, for a quarter of a century, he was one of the most useful and venerated professors. Ten years after Dr. Physick's appointment, we heard him again. Our recollections of him as a lecturer are, that he was concise, plain, unaffected, unambitious, unimaginative, and straight-forward in his style; with so much peculiarity of manner, and such a manifest reliance on facts, as contributed successfully to fix the attention of the class, and impress upon them all he said. In practical surgery he was, unquestionably, a man of great sagacity, and uncommonly correct judgment, and hence he was still more valuable to society as a surgeon, than a teacher. Indeed, he must be regarded as the most original and accomplished surgeon which America has yet produced. His reading does not appear to have been extensive; but what is better than learning, his thinking was patient, profound and accurate. His whole professional character was eminently practical. In manners, dress and address, he was of the old school of gentlemen. In his intercourse with students he was distant, but cherished towards them a kind disposition. His general propriety and rectitude of character were irreproachable. On the whole, the profession in the United States, in his decease, has lost one of its noblest ornaments; and a faithful account of his life and labors would present him as the greatest contributor to practical surgery, which this country has yet produced.

Died, on the 17th December, after an illness of two weeks, Mr. Edward A. Scruggs, a student of medicine in the Cincinnati College, from Huntsville, Alabama, but a native of Virginia. The talents and honorable deportment of Mr. S. had greatly endeared him to his classmates, and such of the professors as had made his acquaintance. D.

